# Variations in accelerometry measured physical activity and sedentary time across Europe – harmonized analyses of 47,497 children and adolescents

**DOI:** 10.1186/s12966-020-00930-x

**Published:** 2020-03-18

**Authors:** Jostein Steene-Johannessen, Bjørge Herman Hansen, Knut Eirik Dalene, Elin Kolle, Kate Northstone, Niels Christian Møller, Anders Grøntved, Niels Wedderkopp, Susi Kriemler, Angie S. Page, Jardena J. Puder, John J. Reilly, Luis B. Sardinha, Esther M. F. van Sluijs, Lars Bo Andersen, Hidde van der Ploeg, Wolfgang Ahrens, Claudia Flexeder, Marie Standl, Holger Shculz, Luis A. Moreno, Stefaan De Henauw, Nathalie Michels, Greet Cardon, Francisco B. Ortega, Jonatan Ruiz, Susana Aznar, Mikael Fogelholm, Andrew Decelis, Line Grønholt Olesen, Mads Fiil Hjorth, Rute Santos, Susana Vale, Lars Breum Christiansen, Russ Jago, Laura Basterfield, Christopher G. Owen, Claire M. Nightingale, Gabriele Eiben, Angela Polito, Fabio Lauria, Jeremy Vanhelst, Charalambos Hadjigeorgiou, Kenn Konstabel, Dénes Molnár, Ole Sprengeler, Yannis Manios, Jaanus Harro, Anthony Kafatos, Sigmund Alfred Anderssen, Ulf Ekelund, On behalf of the Determinants of Diet and Physical Activity knowledge hub (DEDIPAC); International Children’s Accelerometry Database (ICAD) Collaborators, IDEFICS Consortium and HELENA Consortium, L. B. Andersen, S. Anderssen, A. J. Atkin, G. Cardon, R. Davey, U. Ekelund, D. W. Esliger, P. Hallal, B. H. Hansen, K. F. Janz, S. Kriemler, N. Møller, K. Northstone, R. Pate, J. J. Puder, J. Reilly, J. Salmon, L. B. Sardinha, L. B. Sherar, E. M. F. van Sluijs

**Affiliations:** 1 Department of Sports Medicine, Norwegian School of Sport Sciences, PO Box 4014, Ullevål Stadion, 0806 Oslo, Norway; 2 Population Health Sciences, Bristol Medical School, Bristol, UK; 3 Research Unit for Exercise Epidemiology and Centre of Research in Childhood Health, Department of Sports Science and Clinical Biomechanics, University of Southern Denmark, Odense, Denmark; 4 Epidemiology, Biostatistcs and Prevention Institute, University Zürich, Zürich, Switzerland; 5 Centre for Exercise, Nutrition and Health Sciences, University of Bristol, Bristol, UK; 6 Obstetric service, Lausanne University Hospital, Lausanne, Switzerland; 7 Physical Activity for Health Group, School of Psychological Sciences and Health, University of Strathclyde, Glasgow, Scotland; 8 Portugal, Exercise and Health Laboratory, Faculty of Human Kinetics, Universidade de Lisboa, Lisbon, Portugal; 9 Centre for Diet and Activity Research (CEDAR) & MRC Epidemiology Unit, University of Cambridge, Cambridge, UK; 10 Department of Sport, Food and Natural Sciences, Faculty of Education, Arts and Sports, Western Norway University of Applied Sciences, Sogndal, Norway; 11 Department of Public and Occupational Health, Amsterdam Public Health Research Institute, VU University Medical Center, Amsterdam, Netherlands; 12 Leibniz Institute for Prevention Research and Epidemiology – BIPS, Bremen, Germany; 13 Helmholtz Zentrum München, German Research Center for Environmental Health, Institute of Epidemiology, Neuherberg, Germany; 14 GENUD research group, Facultad de Ciencias de la Salud, Universidad de Zaragoza, Insituto Agroalimentario de Aragón (IA2), Instituto de Investigación Sanitaria Aragón (IIS Aragón), Centro de Investigación Biomédica en Red Fisiopatología de la Obesidad y Nutrición (CIBEROBN), Zaragoza, Spain; 15 Department of Public Health and Primary Care, Faculty of Medicine and Health Sciences, Ghent University, Ghent, Belgium; 16 School of Sport Sciences, University of Granada, Granada, Spain; 17 PAFS Research group, Faculty of Sports Sciences, UCLM, Ciudad Real, Spain; 18 Department of Food and Nutrition, University of Helsinki, Helsinki, Finland; 19 Institute for Physical Education and Sport, University of Malta, Msida, Malta; 20 Department of Nutrition, Exercise and Sports Unit for obesity research Faculty of Science, University of Copenhagen, Copenhagen, Denmark; 21 Faculty of Sport, University of Porto, Porto, Portugal; 22 Department of Sport Science, High School of Education, Polytechnic Institute of Porto, Porto, Portugal; 23 Institute of Health & Society and Human Nutrition Research Centre, Newcastle University, Newcastle upon Tyne, UK; 24 Population Health Research Institute, St George’s, University of London, London, UK; 25 Department of Biomedicine and Public Health, School of Health and Education, University of Skövde, Skövde, Sweden; 26 CREA Research Centre for Food and Nutrition, Rome, Italy; 27 National Research Council, Institute of Food Sciences, Avellino, Italy; 28 Inserm, CHU Lille,U995 - LIRIC - Lille Inflammation Research International Center, CIC 1403 – Clinical Investigation Centre, University of Lille, F-59000 Lille, France; 29 Research and Education Institute of Child Health, Strovolos, Cyprus; 30 National Institute for Health Development, Tervise Arengu Instituut, Tallin, Estonia; 31 University of Pecs, Medical Faculty, Pécs, Hungary; 32 Department of Nutrition & Dietetics, Harokopio University, Athens, Greece; 33 Department of Psychology, Estonian Centre of Behavioural and Health Sciences, University of Tartu, Tartu, Estonia; 34 School of Medicine, University of Crete, Heraklion, Greece

## Abstract

**Background:**

Levels of physical activity and variation in physical activity and sedentary time by place and person in European children and adolescents are largely unknown. The objective of the study was to assess the variations in objectively measured physical activity and sedentary time in children and adolescents across Europe.

**Methods:**

Six databases were systematically searched to identify pan-European and national data sets on physical activity and sedentary time assessed by the same accelerometer in children (2 to 9.9 years) and adolescents (≥10 to 18 years). We harmonized individual-level data by reprocessing hip-worn raw accelerometer data files from 30 different studies conducted between 1997 and 2014, representing 47,497 individuals (2–18 years) from 18 different European countries.

**Results:**

Overall, a maximum of 29% (95% CI: 25, 33) of children and 29% (95% CI: 25, 32) of adolescents were categorized as sufficiently physically active. We observed substantial country- and region-specific differences in physical activity and sedentary time, with lower physical activity levels and prevalence estimates in Southern European countries. Boys were more active and less sedentary in all age-categories. The onset of age-related lowering or leveling-off of physical activity and increase in sedentary time seems to become apparent at around 6 to 7 years of age.

**Conclusions:**

Two third of European children and adolescents are not sufficiently active. Our findings suggest substantial gender-, country- and region-specific differences in physical activity. These results should encourage policymakers, governments, and local and national stakeholders to take action to facilitate an increase in the physical activity levels of young people across Europe.

## Introduction

There is compelling evidence that higher levels of physical activity are associated with substantial health benefits in young people [1, 2], these benefits seem to be independent of sedentary time [3]. Young people nevertheless spend a large proportion of their waking hours sedentary and many do not appear to be physically active according to the current public health recommendations [4]. Previous studies examining accelerometer-measured physical activity from a diverse range of European children and adolescents suggest a substantial variation in physical activity levels across studies [4–8]. Much of this variation, however, may be artefactual, explained by differences in the methodologies used to reduce, processing, and analyze the accelerometer data [9]. This limitation can be overcome by combining and reprocessing individual-level data from existing studies in a harmonized and standardized manner. This would provide a more consistent and comprehensive estimate of the levels of physical activity and sedentary time in European youth that could inform public-health policymakers across Europe.

The International Children’s Accelerometry Database (ICAD) [9], has already developed standardized methods to create comparable physical activity variables from more than 20 studies including more than 32,000 participants. Cooper et al. [4] used this database to describe variations in physical activity and sedentary time between seven European countries. Similarly, other large pan-European studies have described objectively measured physical activity patterns in children [8] and adolescents [7] using standardized methods. Results from these studies [4, 7, 8] consistently suggest that boys are more active than girls and that physical activity declines with increasing age. No previous study, however, has attempted to pool and harmonize all available accelerometer-measured physical activity data in European children and adolescents. Results from such a harmonized approach will provide a more comparable estimate of physical activity across studies which can be used to spur policymakers, governments, and local and national stakeholders to take action to facilitate structural changes aimed at increasing physical activity levels. Thus, the aim of this study was to assess the variations in physical activity and sedentary time by place and person in European children and youth. We used a systematic literature search and analysed personal level data using a harmonized approach including studies from 1997 to 2014.

## Methods

### Data sources, literature search and study selection

We identified published studies through a systematic review of six databases (PubMed, PsycINFO, EMBASE, Web of Science, Sport Discus, and Scopus) from database inception through March 16th, 2016. Updating the search through Sept 10th, 2017 did not reveal any new datasets.

The following search terms were used: “physical activity” OR “physical activities” OR “physically active” OR “physical exercise” OR “physical activity level” OR sedentary” OR Sedentari* OR “sitting” OR “physical inactivity” OR “physically inactive” AND “Acceleromet*” OR “activity monitor” OR “motion sensor” OR “actigraph”.

All retrieved records were imported into EndNote X7 (Thomson Reuters, New York). Duplicates were hand-searched and removed. Records were included if they were written in the English language; included European study samples aged 2–18 years; and were cross-sectional studies, prospective cohort studies, or controlled trials that had assessed physical activity objectively using the ActiGraph accelerometer. In addition, studies were only included if they provided data from more than 400 individuals. One author (JSJ) extracted the following information from each eligible article: name of the first author, study location, number of participants, age, and physical activity assessment details.

### Data harmonization and data pooling

We contacted the principal investigators of the studies eligible for inclusion and asked whether they were willing to participate in this study, reminding them once or twice if they did not respond. Data-sharing agreements were subsequently signed for studies that agreed to take part and raw accelerometer data files (e.g. .dat, GT3X) and descriptive data (country, age, sex, height, and weight) were transferred to the analytical team. For those studies already included in ICAD, data were made available according to the ICAD applications and authorship agreement (http://www.mrc-epid.cam.ac.uk/research/studies/icad/).

The included studies had assessed physical activity using both uniaxial and triaxial hip-worn accelerometry (ActiGraph models 7164, GT1M, Actitrainer and GT3x/3X+). For consistency across studies, we extracted data from the vertical axis only, reintegrated all files to 60s epochs, and processed all data according to the suggested settings from ICAD 2.0 (http://www.mrc-epid.cam.ac.uk/research/studies/icad/) using the commercially available software KineSoft (v3.3.80, Loughborough, UK, http://www.kinesoft.org). Non-wear time was defined as 60 min of consecutive zeros allowing for 2 min of non-zero interruptions. To overcome challenges with different wear time protocols we excluded data recorded from 23:59 to 06:00 and all non-wear time, we considered days with ≥480 min of activity recordings as valid. Repeated measurements of a child were regarded as multiple individuals.

Average counts per minute (CPM) were used as a measure of total physical activity. Evenson cut-points [10] were used to define light- (101 to ≥2295), moderate- (≥ 2296 CPM), and vigorous-intensity (≥ 4012 CPM) physical activity. These cut points show the best overall performance across all intensity levels [11] and suggested as the most appropriate cut points for youth [12]. For descriptive purposes, we defined time spent sedentary as all-time (min) spent ≤100 CPM. The numbers of minutes per day in different intensities were determined by summing all minutes where the activity count were equal to and greater than the threshold for that intensity, divided by the number of valid days. Irrespective of age, participants achieving on average ≥ 60 min of MVPA per valid day were defined as being sufficiently physically active.

### Anthropometry

Trained personnel measured height and weight using standardized techniques across studies. We calculated body mass index (BMI) as weight (in kilograms) divided by height (in meters) squared. For descriptive purposes, we further categorized individuals as normal weight, overweight, and obese based on age- and sex-specific cut-offs [13]. A small number of participants was categorized as underweight (8%) and combined with the normal weight group.

Local ethics committee approval, parental/legal guardian consent, and child assent were obtained in all studies.

### Statistical analyses

All analyses were conducted in Stata 13.1 (StataCorp., 2013. Stata Statistical Software: TX: StataCorp LP). Descriptive statistics were used to assess sample characteristics as well as levels of physical activity and sedentary time. Multivariable regression analyses, stratified by children (age < 10 y) and adolescents (≥ 10 y), were used to compare total physical activity levels (CPM), MVPA, and sedentary time between countries and across European regions (i.e. north, west, east and south) as demarcated by the United Nations (https://unstats.un.org/unsd/methodology/m49/). Due to lack of countries (only Hungary) to cover the eastern region we merged west and east to central Europe. Multivariable logistic regression analyses were conducted to estimate the odds ratios (ORs) for those defined as sufficiently physically active across sexes, BMI categories, and regions of Europe. To obtain an overall European weighted prevalence estimate we used prevalence estimates from each country weighted by the square root of number of participants within each country. We performed sensitivity analyses by excluding participants from the two largest cohorts one at a time (ALSPAC and IDEFICS) and subsequently participants from the UK and repeated analyses. As participants were recruited from different studies across different countries, we used “study” as a cluster variable in all models to obtain robust variance estimations. Moreover, sex, age, country, season, study year, ActiGraph model, and wear time (where appropriate) were included as covariates in all analyses. Statistical significance was set at *p* < .05.

#### Role of founding source

The study sponsors were not involved in study design; in the collection, analysis, and interpretation of data; in the writing of the report; and in the decision to submit the paper for publication. The corresponding author had full access to data in the study and had final responsibility for the decision to submit for publication.

## Results

**Fig. 1:**
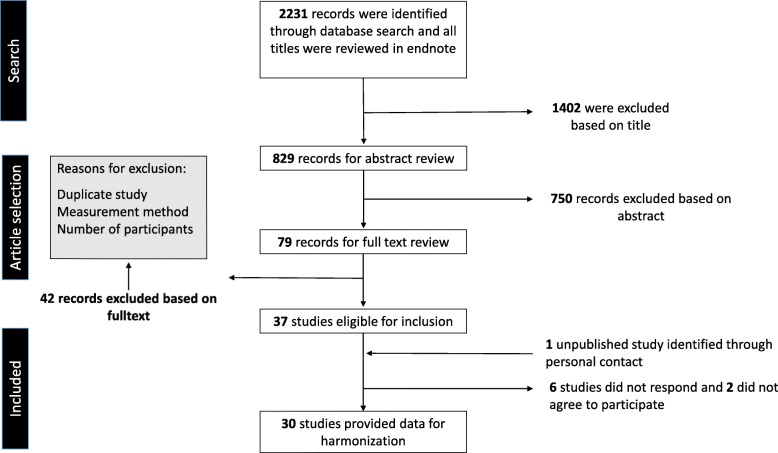
Study selection

In total, 2231 articles were identified by the literature search. We retrieved 79 papers for full-text review, of which 37 studies were identified as eligible for inclusion (Fig. 1). The principal authors of these studies were contacted regarding their willingness to contribute to the harmonized pooled analyses by sharing their data. Twenty-nine [14–37] of the 37 studies’ authors agreed, whereas the remaining eight [38–46] either refused to participate or did not respond to our requests. We additionally obtained data from one previously unpublished Portuguese study. Thus, 30 studies including 18 countries and 51,828 individuals aged 2–18 years were eligible for the harmonized pooled analyses. Of these, accelerometer data were missing from 1879 individuals.

After reanalysis, 48,242 of 49,949 eligible files (96·6%) were deemed valid (Table 1). Reasons for exclusion included: zero days with a wear time of < 480 min and no data on file (*n* = 1534) and monitor malfunction (*n* = 173). In addition, 745 individuals were missing descriptive data (country, sex, age, weight, or height), leaving a total sample of 47,497 individuals included in the present analyses. On average, the included participants provided 5 (1·7 SD) valid days of measurement and 12·9 (1·7 SD) hours of wear time per valid day. Girls were slightly overrepresented (52% of participants), as were data from participants aged 7–15 years (75%) and participants from the UK (39%). Fifteen percent of the sample was classified as overweight and 5% as obese. Supplementary descriptive characteristics of the analytical sample by country are summarized in Additional file 1.

**Table 1:** Studies in alphabetic order: country of origin, design and characteristics of study participants included in the present analyses

Study Name	Yrs	Months	Country	Design	Files^a^	Age (y)	Model	Epoch
ALSPAC	2003–07	All	United Kingdom	Long.	10,426	10–15	7164, 71,256,GT1M	60
Ballabeina Study	2008–09	June-Sept	Swiss	Inter.	998	4–8	GT1M	15
Belgium Pre-School Study	2006;08–09	Oct-March	Belgium	CS	170	3–7	GT1M	15
CHASE	2006–07	Jan-Feb	United Kingdom	CS	2011	9–10	GT1M	15
COSCIS	2001–05	Oct-May	Denmark	Inter.	1146	6–11	7164	60
EYHS (Denmark)	1997–98; 2003–04	All	Denmark	Long.	1715	8–18	7164	60
EYHS (Estonia)	1998–99	Aug-May	Estonia	CS	660	8–17	7164	60
EYHS (Norway)	1999–00	Feb-Oct	Norway	CS	387	9–10	7164	60
EYHS (Portugal)	1999–00	Jan-July	Portugal	Long.	1357	8–18	7164	60
EYHS SPAIN	2008–10	–	SPAIN	CS	447	8–10	GT1M	15
GINI	2011–14	All	Germany	CS	1220	14–17	GT3X	60
Helena	2006–07	All	Austria, Belgium, France, Germany, Greece, Hungary, Italy, Spain, Sweden	CS	2755	13–17	GT1M	15
IDEFICS	2007–2010	Sept-May	Italy, Estonia, Cyprus, Belgium. Sweden, Germany, Hungary, Spain	Long	7104	2–9	GT1M/Actitrainer	15,60
ISCOLE	2011–13	All	Finland	CS	531	9–11	GT3X	15
KISS	2005–06	May-Nov	Swiss	Inter.	889	6–14	7164, GT1M	60
LISA	2011–14	All	Germany	CS	429	14–16	GT3X	60
MAGIC	2006–07	Nov-May	United Kingdom	CS	434	3–4	7164	60
MAL-TA	2012	Jan-May	Malta	CS	859	10–11	GT3X	10
Odense Preschool	2009	May–June	Denmark	CS	527	5–6	GT1M/GT3X	10
OPUS	2011	Aug-Nov	Denmark	Long	705	8–11	GT3X (+)	60
PANCS	2005–06	All	Norway	CS	2031	9–15	7164	10
PEACH	2006–09	Sept-July	England	Long.	2088	10–13	GT1M	15
Portugal	2008–09	All	Portugal	CS	2557	10–18	GT1M	15
Prestyle	2009	–	Portugal	CS	567	3–6	GT1M	5
ProActive	2012–13	–	United Kingdom	CS	1207	10–11	GT3X	15
Portugal	2010–11	Sept-June	Portugal	CS	660	11–12	GT1M	30
SPACE	2010	Apr-June	Denmark	Inter.	1274	11–13	GT3X	30
SPEEDY	2007	Feb-July	United Kingdom	CS	1992	9–11	GT1M	5
The Belgian Environmental PA study in Youth	2008–09	Oct-May	Belgium	CS	606	13–15	GT1M	60
The Gateshead Millennium Study	2006–07	Oct-Dec	United Kingdom	Cross	478	6–8	GT1M	15
The Gateshead Millennium Study	2006–07	Oct-Dec	United Kingdom	Cross	478	6–8	GT1M	15

*ALSPAC* Avon Longitudinal Study of Parents and Children; *CHASE* Child Heart And Health Study in England; *GINIplus* German Infant Study on the influence of Nutrition Intervention PLUS environmental and genetic influences on allergy development; *HELENA* Healthy Lifestyle in Europe by Nutrition in Adolescence Study; *IDEFICS* Identification and prevention of Dietary- and lifestyle-induced health EFfects In Children and infantS; *ISCOLE* The International Study of Childhood Obesity, Lifestyle and the Environment KISS, Kinder-Sportstudie; *LISA* Influence of Life-style factors on the development of the Immune System and Allergies in East and West Germany; *MAGIC* Movement and Activity Glasgow Intervention in Children; *MAL-TA* Movement, Activity and Lifestyle- tweens in action; *PEACH* Personal and Environmental Associations with Children’s Health; *SPEEDY* Sport, Physical activity and Eating behaviour: Environmental Determinants in Young people; *SPACE*,

^a^Valid files included in analyses

### Comparison across sex and age

**Fig. 2:**
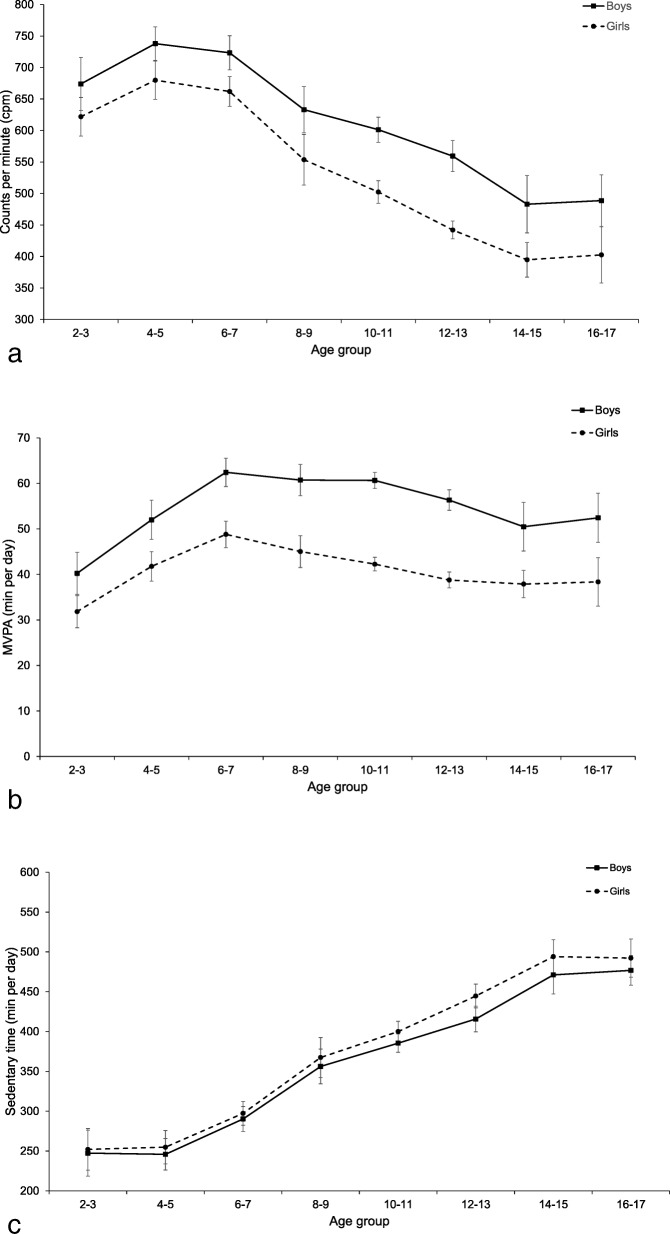
**a**-**c**. Predicted physical activity level (95% CI) by age and sex for **a**) total physical activity (CPM); **b**) time spent in moderate to vigorous (MVPA) and **c**) time spent sedentary (SED). All estimates are adjusted for wear time (b and c), country, season, study year and ActiGraph models

In general, participants spent 49·2% of their measured time sedentary, 44·4% being light physically active, and 6·4% being physically active with moderate to high intensity (MVPA). In both age groups, boys were more active (children: 13 min MVPA/day, 95% CI: 12, 14; adolescents: 17 min/day, 95% CI: 16, 18) and spent less time sedentary compared to girls (children: 8 min/day, 95% CI: 6, 11; adolescents 22 min/day, 95% CI: 19, 25 for adolescents). Average CPM as well as intensity-specific activity (MVPA and sedentary time) were significantly associated with age. Categorizing male and female participants into eight age categories (2–3, 4–5, 6–7, 8–9, 10–11, 12–13, 14–15, and 16–17) suggested substantial age group differences in average CPM. Counts per minute were highest at ages 4–5 years and were then progressively lower in every age group until ages 14–15 years, with an average category-to-category difference of 54 CPM. The most pronounced difference was observed between ages 6–7 and 8–9 years (− 101 CPM, 95% CI: − 197, − 6). Time spent in MVPA was highest at ages 6–7 years and was progressively lower by increasing age groups, with an average difference of 2·8 min/day by age category. Time spent sedentary (min/day) increased progressively from ages 4–5 years to 16–17 years (Fig. 2, a–c). Females, overweight and obese participants, showed significantly lower odds of being categorized as sufficiently physically active (Table 3).

### Comparison across countries

**Fig. 3:**
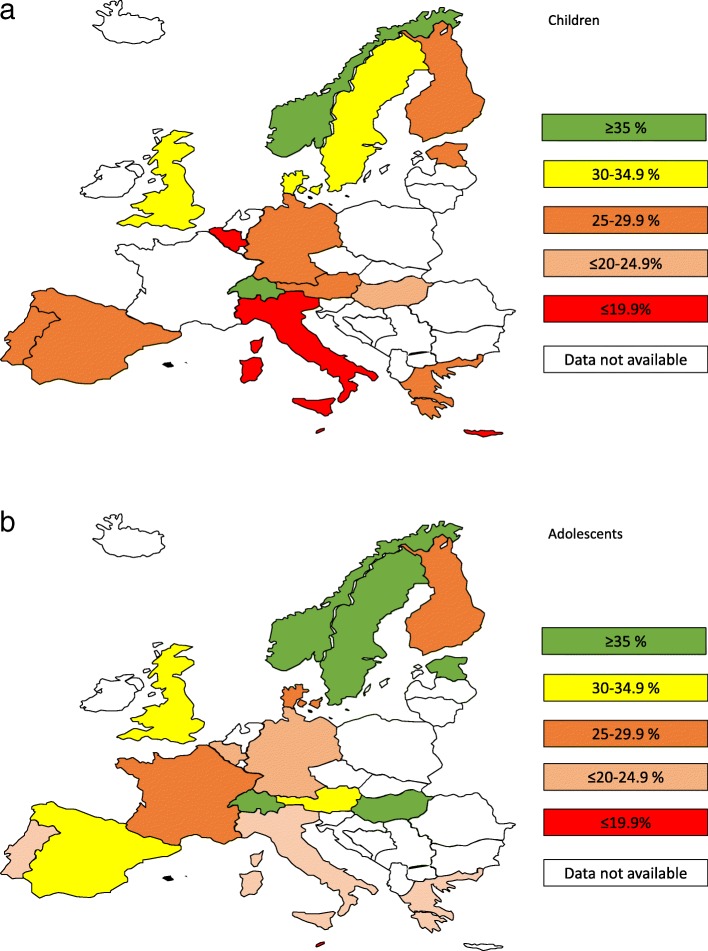
**a** Prevalence categories (≤ 19·9%, 20·0-24·9%, 25·0-29·9%, 30·0-34·9% and ≥35 %) of children being sufficiently physical active by country. **b** Prevalence categories (≤ 19·9%, 20·0-24·9%, 25·0-29·9%, 30·0-34·9% and ≥35 %) of adolescents being sufficiently physical active by country

The prevalence of being categorized as sufficiently active (i.e. accumulating an average of ≥60 min/day of MVPA) by region, country, and age group is shown in Table 2 and Fig. 3a and b. Overall, weighted estimates suggested that 29% (95% CI: 25, 33) of children and 29% (95% CI: 25, 32) of adolescents were categorized as sufficiently physically active as defined by an average of at least 60 min MVPA per day. In sensitivity analyses, excluding participants from the two largest cohorts one at a time (ALSPAC and IDEFICS) and subsequently participants from the UK had only a minor impact on prevalence estimates (a 1–3% reduction in prevalence, data not shown). Across regions, the highest prevalence of sufficiently active was observed in Northern European countries with significant lower estimates in Southern-European countries. The prevalence of those defined as sufficiently active was highest living in Northern Europe (31%) compared to Central Europe (26%) and those living in Southern Europe (23%). As illustrated in Table 2, there were substantial differences in prevalence estimates across countries, with the highest estimates recorded in Swiss children (38, 95% CI: 25, 51) and Swiss adolescents (43, 95% CI: 37, 48). The lowest prevalence estimates were observed participants in Southern European countries, where only 13% (95% CI: 9, 16) of Cypriot children and 14% of Maltese adolescents were sufficiently active.

**Table 2:** Prevalence (95% CI) of for being categorized as sufficiently physically active by European region, country and age group

European region	Overall region	Country within region	Children (2–9.9 y)	Adolescents (≥10–18 y)
North (*n* = 28,988)	31 (29,34)	Norway	37 (26, 49)	34 (32,37)
		Sweden	33 (28,39)	38 (31,44)
		Denmark	32 (24,41)	29 (21,37)
		Finland	25 (11,38)	29 (15,43)
		Estonia	28 (23,32)	40 (29,52)
		UK	31 (21,40)	30 (27,32)
Central (*n* = 9287)	26 (20,32)	France	N/A	28 (23,33)
		Germany	33 (28,38)	24 (10,38)
		Austria	N/A	34 (27,40)
		Swiss	38 (25, 51)	43 (37,48)
		Belgium	18 (10,26)	20 (16,23)
		Hungary	22 (19,25)	38 (31,46)
South (*n* = 9222)	23 (20,27)	Portugal	25 (21,29)	24 (19,29)
		Spain	25 (21,28)	33 (29,37)
		Italy	N/A	21 (17,26)
		Malta	N/A	14 (10,19)
		Cyprus	13 (9,16)	N/A
		Greece	N/A	27 (22,33)

All estimates are adjusted for sex, age, wear time, country, season, study year and ActiGraph models. Study used as cluster variable

Average CPM and time spent sedentary and in MVPA varied substantially between countries in both younger and older age groups (Additional file 3). Among children, the difference between the least active (Cyprus) and most active (Norway) countries for total PA was 169 CPM (95% CI: 55, 283), which corresponds to a difference in accelerometer output of 24%. In adolescents, similar differences (141 CPM, 95% CI: 73, 210) were observed between the most active (Norwegians) and least active (Maltese). Northern European countries (Norway, Estonia, the UK, Sweden, and Denmark) recorded the highest levels of total PA regardless of age. We observed similar differences between countries when we repeated the analyses using time spent (minutes/day) in MVPA and sedentary time as outcomes. In children, participants from Cyprus spent on average 19 (95% CI: 8, 31) fewer minutes in MVPA per day compared to their Norwegian counterparts, whereas in the oldest age group, the Maltese adolescents spent on average 25 (95% CI: 16, 33) fewer minutes in MVPA compared to the Swiss participants. The Finnish children and Belgian adolescents spent the most amount of time sedentary.

We observed a significant north-south MVPA gradient (*p* = .001), with Southern European and Central European participants having an odds ratio for being sufficient physical active at 0·63 and 0·75, respectively, compared to participants from the Northern Europe (Table 3). Southern European participants spent an average of 5 min (95% CI: 2, 9) per day in MVPA less than participants from Northern Europe. There was no difference in time spent in MVPA between Northern and Central European participants. The same significant north-south gradient was also evident for total physical activity and sedentary time, showing average differences of 34 CPM (95% CI: 18, 49) and 15 min sedentary time per day (95% CI: 6, 22) when moving from one region to another (Additional file 2).

**Table 3:** Odds ratio (95% CI) for being categorized as physically active by sex, weight status and European region

	Total		Children		Adolescents	
	%	OR (95%CI)	%	OR (95%CI)	%	OR (95%CI)
Overall^a^		N/A	28·7		28·9	
Sex						
Male (ref)	40·6	1·00	38·7		41·8	
Female	18·0	**0·30 (0·26, 0·35)**	20·2	**0·37 (0·31, 0·44**)	16·6	**0·26 (0·23, 0·29)**
Weight status						
Normal (ref)	30·5	1·00	30·8	1·00	30·5	1·00
Overweight	23·6	**0·68 (0·61, 0·76)**	24·6	**0·71(0·61, 0·82)**	22·4	**0·62 (0·54, 0·72)**
Obese	19·3	**0·51 (0·42, 0·63)**	18·6	**0·48 (0·36, 0·63)**	18·5	**0·48 (0·38, 0·61)**
European region						
North (ref)	31·4	1·00	31·3	1·00	29·9	1·00
Central	26·0	0·75 (0·51, 1·01)	29·4	0·90 (0·63, 1·27)	28·5	0·93 (0·56, 1·54)
South	23·2	**0·63 (0·50, 0·79)**	23·8	**0·65 (0·51, 0·83)**	23·4	**0·69 (0·51, 0·93)**

All estimates are adjusted for sex, age, wear time, country, season, study year and ActiGraph models. Study used as cluster variable. ^a^In overall estimates each country weighted by the square root of participants within each country

## Discussion

These analyses—including data from more than 47,000 young people across 18 European countries—indicate that overall physical activity, time spent in MVPA, sedentary time, and prevalence of being sufficiently physical active differ substantially between countries and regions. We observed a north-south gradient showing lower physical activity levels and more time spent sedentary among Southern European participants. Indeed, the prevalence of those defined as sufficiently active was lower among those living in Southern Europe (23%) compared to those living in Northern Europe (31%).

Differences in physical activity and sedentary time between countries have consistently been described in the literature [5, 6]; it has been proposed, however, that much of this variation is likely due to methodological differences related to the reduction, processing, and analysis of accelerometer data. Our harmonized analyses allowed us to compare physical activity levels across countries with greater accuracy and precision in a larger and more diverse European population than has previously been possible [4, 7, 8]. The substantial differences between countries are similar to previous results from other pan-European cohorts including device-based measures of activity [4, 7, 8]. The observed 30–35% difference in total physical activity level (CPM) between the most and least active countries indicates substantial variation in physical activity levels in European youth.

Based on analyses stratified by region (north, central, and south; see Additional file 2), we observed higher levels of physical activity (CPM and MVPA) and lower time spent sedentary among Northern European individuals compared to those living in Southern Europe. This north-south gradient, where individuals living in Southern Europe were 64% less likely to be classified as sufficiently physically active compared to their peers living in Northern Europe. This pattern was consistent across age and independent of body mass index BMI (data not shown). Based on pan-European data, Ruiz et al. [7] observed differences in physical activity patterns in central-Northern versus Southern European adolescents. The differences between regions, however, were less pronounced compared to those reported here, possibly explained by a smaller sample size, more narrow age span (aged 10–18 years) and only included data from nine countries. A similar north-south gradient have also been observed in a study by Konstabel et al. [8] but only including children in pre- and primary school from eight countries. Thus, our results provide a more comprehensive description of physical activity levels among European children and adolescents which suggests that regional differences observed are unlikely explained by differences in methods used. Possible explanations for the apparent north–south gradient remain unclear and further studies including harmonized data on objectively assessed physical activity in combination with individual, social, and environmental data on determinants of physical activity (and sedentary time) are needed [4, 5]—for example, cultural differences and the extent to which physical activity policies are developed and prioritized within countries may influence physical activity levels. Thus, the results presented are of importance for regional, national, and European policymakers in facilitating implementations of programs aimed at increasing physical activity in all European children and adolescents.

In total, 29% of the study population was categorized as sufficiently physically active. In comparison, Cooper et al. [4] reported a significantly lower prevalence estimate based on ICAD data, with only 9% of boys and 2% of girls achieving the recommended activity levels. Discrepancies between estimates are not only explained by the different MVPA cut-point used, but also the interpretation of guideline adherence. Cooper et al. [4] used conservative criteria requiring participants to accumulate ≥ 60 min of MVPA on every measured day, whereas we used more liberal criteria in which accumulating on *average* ≥ 60 min of MVPA per day during the measurement period was deemed sufficient. Both guideline-adherence interpretations have been used in multi-national studies in children and adolescents [4, 7, 8, 47, 48] and highlights one of the major challenges when comparing data using different interpretations. Interestingly, Cooper et al. [4] also provided data using a more liberal interpretation, showing that ≥60 min of MVPA was accumulated on 46% of days for boys and 22% for girls. These results, although not directly comparable, are more in line with our own estimates; regardless, we report that at least two-thirds of European children and adolescents are insufficiently active and should be of concern for public health authorities.

Our observations corroborate previous findings [4, 7, 8] showing that boys are more active than girls and that differences in activity increase with age. The cross-sectional age-related negative trend in physical activity observed in the present study is a commonly reported finding; however, some discrepancies exist regarding the onset of this trend [4, 49, 50]. In cross-sectional analyses, Cooper et al. [4] observed gradually lower activity levels starting from ages 5–6, whereas a previous systematic review and pooled analyses from longitudinal studies [49] concluded that physical activity declines with the onset of adolescence. However, only 2 of the 26 studies used device-based measures and most studies were conducted before the year 2000. Thus, the generalizability to contemporary populations might be questionable. Farooqa et al. [50] recently presented longitudinal analyses from the Gateshead Millennium Cohort Study reporting a marked decline in physical activity during childhood (from age 7 years to 15 years).

In this line, we observed that the onset of age-related lowering or leveling-off of physical activity seems to occur at around 6 to 7 years of age. Taken together, the transition between early childhood (preschool) and childhood (primary school) appears to be a critical period where interventions aimed at preventing a decline in physical activity are important; nonetheless, there is still a need for more longitudinal cohort studies describing changes in device-based measured physical activity across childhood, adolescence, and even the transition to young adulthood.

### Strengths and limitations

The main strength of this study is the use of individual-level accelerometer data, harmonized (i.e. cleaned, processed, and re-analyzed) in a consistent manner, across studies. This is by far the largest harmonized individual physical activity dataset including a wide age range (2–18 years) and individuals from 18 European countries.

Some limitations should also be acknowledged. First, differences in recruitment and sampling methodology within cohorts and between studies likely limit the representativeness to fully reflect the true prevalence of physical activity. Thus, it is possible that the observed differences between countries are due to incorrect representation of the actual demographic distribution within a country. For example, it seems that participants in some studies are less likely to be overweight or obese than the general population [51]. Given the inverse association between BMI and physical activity, we cannot rule out that these participants may be more active than the general population; thus, the true physical activity levels and physical activity prevalence estimates may be somewhat overestimated in this study. The choice to only include studies with more than 400 individuals could be considered arbitrary, however this was done to increase the possibility of representativeness for each country. Second, differences in data collection procedures may have influenced the results and thus may partly explain the observed differences between countries. For instance, some studies used a 24-h protocol, a four-day protocol, whereas others assessed physical activity for at least 7 days and the sampling of weekdays and weekend days may be different within and between studies. To overcome some of these limitations, we excluded data recorded from 23:59 to 06:00, adjusted for monitor wear time and used “study” as a cluster variable in all analyses. However, we cannot rule out that for some individuals, in those studies employing a 24 h- hour protocol, sedentary time might have been overestimated.

The accelerometer thresholds and the selection of epoch length should also be considered. It is well-established that these decisions have a substantial impact on physical activity intensity outcomes (e.g. time spent in MVPA) when assessing adherence to physical activity recommendations. Thresholds for intensity levels used in the present study are in line with a previous multi-country study [4] and have been shown to provide valid estimates for children and adolescents [10]. Age-specific thresholds have been developed for toddlers aged 2 to 5 years [52, 53]. These thresholds are, however, developed based on shorter epochs (5–15 s) and it is unclear whether these are applicable to the 60-s epoch used in the analyses herein. Thus, we have not included age-specific cut points for those under the age of 5 years, which might result in underestimation of time spent in MVPA for this age-group. Anyhow, sensitivity analyses excluding all participants < 5 years of age (*N* = 3348, 7% of the total sample) revealed only minor changes in the prevalence estimates, indicating that our main conclusion, suggesting a northern-southern gradient, is valid (Additional file 5). A short epoch length (e.g. 10 s) is the preferred option in young people [12]; however, we used a 60-s epoch for the purpose of data harmonization, as some of the studies we included collected their data using this epoch length. This may lead to misclassification of MVPA as light physical activity, less time accumulated in MVPA and subsequently an underestimation of the prevalence of being categorized as physically active. Including cohorts spanning a relatively long time period (1997–2015) introduces a potential bias due to the use of different ActiGraph models. To date, newer generations of ActiGraphs, i.e. from the GT1M and forward, can be compared and used interchangeably [54] but comparability with the older 7164 model is unclear [55]. Nevertheless, including “ActiGraph model” as a covariate had a substantial impact on our results, as the regression models showed higher physical activity outputs (CPM and MVPA) with the oldest model (7164) compared to the newer models (GT1M, GT3X) (Additional file 4). However, the study design of the present study does not allow exploring to what extent differences in physical activity outputs could be explained by model used. Thus, we cannot rule out that adjustment for model might have led to both underestimation (7164) and overestimation (GT1M, GT3X) of the true physical activity level. Nonetheless, sensitivity analyses excluding “ActiGraph model” from the regression model did not change the ranking of countries or regions for any of the outcomes. Taken together, the present results should be interpreted in light of the abovementioned limitations.

Third, a known limitation of accelerometers is their inability to capture certain movements, such as cycling, which may differ substantially between countries. There is some evidence suggesting that that physical activity during cycling as transportation is substantially underestimated when using accelerometers [56]. Thus, in countries with a high prevalence of cycling for transportation such as Belgium and Denmark, physical activity levels may therefore have been underestimated in these countries. In addition, hip-mounted accelerometers cannot measure posture and distinguish between sitting and standing, thus our estimates of sedentary time may include both sitting and standing still. Fourth, we defined prevalence of sufficiently active as an average of 60 min per day due to differences between studies in protocols. Thus, our prevalence estimates may be overestimated. Furthermore, we did not distinguish between toddlers (1–5 years old), children, and adolescents when applying our definition of being sufficiently active. Some countries have proposed separate physical activity guidelines for children < 5 years stating that they should be physically active (at any intensity) for at least 180 min (3 h) daily, spread throughout the day. Although, the evidence for a causal association between physical activity and health in children and adolescents is limited and obtained from predominantly observational research compared with that for adults, there is growing evidence that MVPA is associated with greater health benefits than lower intensity activity [2]. Thus, we consider time spent in MVPA more relevant when defining sufficiently active individuals, although acknowledging that this is affected by the choice of cut points to define MVPA.

Fifth, it is well-known that children’s activity levels exhibit a seasonal pattern [57], and weekly pattern [58] with lower levels during the winter months when cold weather and reduced daylight is suggested to reduce physical activity [59]. Thus, we cannot rule out that seasonality, weekly pattern, weather, and temperature may have affected our results; on the other hand, the majority of the included studies collected data over several months covering multiple seasons, and we included season as a covariate in all analyses.

Finally, the relatively large timespan between the earliest (1999) and latest (2016) data collections needs to be considered; one may speculate that the observed differences between countries and European regions could be explained by secular trends with decreased physical activity levels over time. However, our data did not reveal any significant association between any physical activity outcome and study year (data not shown) and there is little evidence for any secular trends in physical activity during the last decades [60, 61].

Although the present harmonized individual-level accelerometer data does increase comparability between studies, the abovementioned limitations highlight the need for more standardized data collection, including a setup for large pan-European surveillance of physical activity and sedentary time using accelerometry.

## Conclusion

Our pan-European data show that more than two-thirds of European youth can be categorized as insufficiently active. Our findings also suggest substantial country- and region-specific differences in physical activity reaching up to 30–35% differences in total physical activity (CPM) between the least and most active countries, with a clear trend of lower levels in Southern compared to Northern regions, i.e., 23% vs. 31% of participants meeting the physical activity recommendations respectively. These results should urge policymakers, governments, and local and national stakeholders to immediately facilitate structural and political changes to promote physical activity in European youth.

## Supplementary information

**Additional file 1.** Descriptive characteristics (mean, SD) of study participants by country. This table describes proportion of boys and girls, age and weight status within each country

**Additional file 2.** Accelelloremeter-assessed physical activity and sedentary time by region for the total sample and based on ages < 10 and ≥ 10. This table describes physical activity and sedentary time by region North, West and East

**Additional file 3.** Predicted time spent per day in total physical activity, moderate to vigorous PA and sedentary by country and stratified by children and adolescents.

**Additional file 4.** Physical activity outputs (CPM and MVPA) by “ActiGraph model”.

**Additional file 5.** Odds ratio (95% CI) for being categorized as physically active by European region excluding participants < 5 years (*n* = 3348)

## Data Availability

The datasets during and/or analyzed during the current study are available from the corresponding author on reasonable request.
