# Supplementary material for: Variations in accelerometry measured physical activity and sedentary time across Europe – harmonized analyses of 47,497 children and adolescents
Source: Int J Behav Nutr Phys Act. 2020 Mar 18;17:38. doi: 10.1186/s12966-020-00930-x (PMC7079516; doi:10.1186/s12966-020-00930-x)
Supplement: Supplementary file 1 — Additional file 1. Descriptive characteristics (mean, SD) of study participants by country. This table describes proportion of boys and girls, age and weight status within each country [file 12966_2020_930_MOESM1_ESM.docx]

**Additional file 1*.* Descriptive characteristics (mean, SD) of study participants by country.**

| **Country** | **N** | **Sex (%)** | | **Age** | **BMI** | **Weight status (%)** | | | |
| --- | --- | --- | --- | --- | --- | --- | --- | --- | --- |
|  |  | Male | Female |  |  | **Underweight** | **Normal** | Overweight | Obese |
| Greece | 382 | 45 | 55 | 14·2 (1·1) | 22·2 (3·9) | **4** | **64** | 21 | 11 |
| United Kingdom | 18,356 | 48 | 52 | 11·2 (2·3) | 18·9 (3·5) | **8** | **70** | 18 | 4 |
| Germany | 3145 | 49 | 51 | 11·9 (4·6) | 19·1 (3·6) | **9** | **75** | 12 | 4 |
| Belgium | 1601 | 48 | 52 | 10·9 (4·6) | 18·4 (3·6) | **9** | **78** | 10 | 3 |
| France | 223 | 42 | 58 | 14·3 (1·0) | 20·5 (3·3) | **9** | **74** | 14 | 3 |
| Hungary | 2258 | 49 | 51 | 7·8 (3·4) | 17·0 (3·3) | **14** | **68** | 12 | 6 |
| Italy | 195 | 59 | 41 | 14·6 (1·1) | 21·6 (3·8) | **7** | **63** | 24 | 6 |
| Sweden | 881 | 49 | 51 | 8·4 (4·1) | 17·0 (2·9) | **11** | **79** | 9 | 1 |
| Austria | 217 | 48 | 52 | 15·0 (1·2) | 21·5 (3·3) | **5** | **74** | 16 | 5 |
| Spain | 2111 | 50 | 50 | 8·0 (3·7) | 17·7 (3·1) | **7** | **69** | 15 | 6 |
| Denmark | 5258 | 47 | 53 | 10·5 (3·0) | 17·9 (3·0) | **8** | **78** | 12 | 2 |
| Estonia | 1579 | 47 | 53 | 8·4 (4·2) | 17·0 (2·8) | **12** | **77** | 9 | 2 |
| Cyprus | 545 | 51 | 49 | 6·3 (1·2) | 16·5 (2·5) | **12** | **66** | 14 | 8 |
| Portugal | 5138 | 48 | 52 | 11·5 (3·5) | 19·4 (3·7) | **7** | **68** | 19 | 6 |
| Malta | 851 | 49 | 51 | 10·7 (0·3) | 19·6 (4·3) | **8** | **57** | 23 | 12 |
| Swiss | 1843 | 51 | 49 | 7·5 (2·6) | 16·5 (2·4) | **10** | **77** | 10 | 3 |
| Norway | 2386 | 52 | 48 | 11·6 (2·8) | 18·6 (3·3) | **7** | **77** | 12 | 4 |
| Finland | 529 | 47 | 53 | 10·0 (0·4) | 17·8 (2·7) | **5** | **75** | 17 | 3 |
| ***TOTAL*** | 47,497 | 48 | 52 | 10·6 (3·4) | 18·6 (3·5) | **8** | **72** | 15 | 5 |
