# Supplementary material for: Variations in accelerometry measured physical activity and sedentary time across Europe – harmonized analyses of 47,497 children and adolescents
Source: Int J Behav Nutr Phys Act. 2020 Mar 18;17:38. doi: 10.1186/s12966-020-00930-x (PMC7079516; doi:10.1186/s12966-020-00930-x)
Supplement: Supplementary file 2 — Additional file 2. Accelelloremeter-assessed physical activity and sedentary time by region for the total sample and based on ages < 10 and ≥ 10. This table describes physical activity and sedentary time by region North, West and East [file 12966_2020_930_MOESM2_ESM.docx]

**Additional file 2. Accelerometer -assessed physical activity and sedentary time by region for the total sample and based on ages <9.9 and ≥10.**

|  |  | **Physical activity indices** | | | | | |
| --- | --- | --- | --- | --- | --- | --- | --- |
|  | **Region** | **Total PA (average cpm)** | **(95% CI)** | **MVPA (min/day)** | **(95% CI)** | **SED (min/day)** | **(95% CI)** |
| **Total** |  |  |  |  |  |  |  |
|  | North | 582 | (562,603) | 51 | (49,53) | 372 | (367,377) |
|  | Central | 549 | (526,572) | 47 | (43,52) | 391 | (384,399) |
|  | South | 513 | (496,531) | 46 | (43,48) | 401 | (384,417) |
| **Children** |  |  |  |  |  |  |  |
|  | North | 665 | (639,691) | 51 | (48,54) | 297 | (290,304) |
|  | Central | 636 | (620,651) | 49 | (46,53) | 306 | (302,310) |
|  | South | 589 | (575,604) | 46 | (44,48) | 315 | (305, 325) |
| **Adolescents** |  |  |  |  |  |  |  |
|  | North | 532 | (512,552) | 50 | (48,52) | 421 | (413,428) |
|  | Central | 489 | (449,530) | 50 | (44,56) | 446 | (428,464) |
|  | South | 463 | (443,484) | 46 | (43,49) | 454 | (429,479) |

All estimates are adjusted for wear time (not cpm), country, season, study year and ActiGraph models. Study used as cluster variable. **Countries representing parts of Europe; North (Norway, Sweden, Denmark, Finland, Estonia and UK); Central (France, Germany, Austria, Swiss, Belgium and Hungary); South (Portugal, Spain, Italy, Malta, Cyprus and Greece).**
