# Supplementary material for: Variations in accelerometry measured physical activity and sedentary time across Europe – harmonized analyses of 47,497 children and adolescents
Source: Int J Behav Nutr Phys Act. 2020 Mar 18;17:38. doi: 10.1186/s12966-020-00930-x (PMC7079516; doi:10.1186/s12966-020-00930-x)
Supplement: Supplementary file 3 — Additional file 3. Predicted time spent per day in total physical activity, moderate to vigorous PA and sedentary by country and stratified by children and adolescents. [file 12966_2020_930_MOESM3_ESM.docx]

**Additional file 3. Predicted total physical activity level (average CPM) and time spent per day in moderate to vigorous physical activity and sedentary (min per day) by country and stratified by children and adolescents.**

| **Country** | **Children (2-9,9 y)** | | | **Adolescents (≥ 10-18y)** | | |
| --- | --- | --- | --- | --- | --- | --- |
|  | CPM (95%CI) | MVPA (95%CI) | SED (95%CI) | CPM (95%CI) | MVPA (95%CI) | SED (95%CI) |
| Austria |  |  |  | 511 (466, 556) | 54·3 (49·5, 59·2) | 456 (436, 475) |
| Belgium | 600 (535, 664) | 43·0 (36·9, 49·2) | 296 (279, 314) | 444 (408, 480) | 42·7 (38·5, 47·0) | 474 (460, 489) |
| Cyprus | 536 (498, 575) | 36·8 (32·8, 40·8) | 316 (304, 327) |  |  |  |
| Denmark | 675 (595,755) | 51·7 (44·3, 59·2) | 300 (277, 323) | 525 (466, 584) | 46·6 (40·2, 53·0) | 428 (409, 447) |
| Estonia | 646 (605, 686) | 49·5 (44·8, 54·2) | 288 (268, 307) | 576 (499, 652) | 60·7 (52·1, 69·2) | 409 (365, 453) |
| Finland | 587 (463, 710) | 46·0 (34·2, 57·8) | 338 (296, 381) | 484 (390, 579) | 47·9 (37·5, 59·4) | 419 (386, 453) |
| France | N/A | N/A | N/A | 465 (426, 504) | 49·4 (45·3, 53·5) | 471 (455, 487) |
| Germany | 691(657, 725) | 51·4 (47·9, 54·8) | 282 (269, 294) | 474 (388, 560) | 46·7 (36·5, 56·8) | 449 (411, 486) |
| Greece | N/A | N/A | N/A | 457 (417, 498) | 45·5 (41·1, 49·9) | 458 (442, 474) |
| Hungary | 579 (549, 610) | 44·3 (41·2, 47·4) | 322 (312, 332) | 527 (485, 569) | 56·0 (51·0, 61·0) | 452 (434, 470) |
| Italy | N/A | N/A | N/A | 453 (412, 494) | 46·3 (41·9, 50·6) | 465 (447, 482) |
| Malta | N/A | N/A | N/A | 438 (378, 498) | 36·3 (29·8, 42·8) | 402 (373, 430) |
| Norway | 706 (627, 784) | 56·3 (48·1, 64·4) | 296 (269, 322) | 579 (558, 601) | 54·3 (51·8, 56·8) | 414 (405, 426) |
| Portugal | 589 (565, 614) | 46·7 (43·8, 49·5) | 323 (301, 345) | 463 (429, 496) | 47·0 (43·9, 50·1) | 472 (454, 489) |
| Spain | 598 (569, 626) | 47·7 (44·8, 50·6) | 305 (296, 315) | 486 (431, 541) | 51·8 (48·6, 55·1) | 461 (407, 516) |
| Sweden | 692 (654, 730) | 52·1 (48·4, 55·8) | 279 (266, 291) | 528 (484, 572) | 53·8 (49·1, 58·6) | 453 (435, 472) |
| Swiss | 672 (636, 709) | 55·2 (45·5, 64·8) | 300 (288, 312) | 552 (532, 571) | 60·9 (56·8, 65·1) | 414 (403, 425) |
| UK | 651 (596, 706) | 49·8 (43·6, 56·0) | 307 (293, 321) | 534 (513, 554) | 50·2 (48·1, 52·3) | 416 (407, 425) |

All estimates are adjusted for wear time (not cpm), country, season, study year and ActiGraph models. Study used as cluster variable.CPM: count per minute; MVPA: moderate to vigorous physical activity; SED: sedentary time.
