# Supplementary material for: Variations in accelerometry measured physical activity and sedentary time across Europe – harmonized analyses of 47,497 children and adolescents
Source: Int J Behav Nutr Phys Act. 2020 Mar 18;17:38. doi: 10.1186/s12966-020-00930-x (PMC7079516; doi:10.1186/s12966-020-00930-x)
Supplement: Supplementary file 4 — Additional file 4. Physical activity outputs (CPM and MVPA) by “ActiGraph model”. [file 12966_2020_930_MOESM4_ESM.docx]

**Additional file 4.** Physical activity outputs (CPM and MVPA) by "ActiGraph model"

|  | Average cpm | MVPA (min/day) |
| --- | --- | --- |
| **CSI 7164** | 585 (581, 589) | 56.3 (55.8, 56.8) |
| **GT1M - GT3X** | 548 (545, 551) | 44.8 (44.4, 45.1) |

All estimates are adjusted for study year.
