# Supplementary material for: Variations in accelerometry measured physical activity and sedentary time across Europe – harmonized analyses of 47,497 children and adolescents
Source: Int J Behav Nutr Phys Act. 2020 Mar 18;17:38. doi: 10.1186/s12966-020-00930-x (PMC7079516; doi:10.1186/s12966-020-00930-x)
Supplement: Supplementary file 5 — Additional file 5. Odds ratio (95% CI) for being categorized as physically active by European region excluding participants < 5 years (n = 3348) [file 12966_2020_930_MOESM5_ESM.docx]

**Additional file 5.** Odds ratio (95% CI) for being categorized as physically active by European region excluding participants <5 years (n=3348)

|  | Total |  | **Children (5-9,9y)** |  | **Adolescents (≥ 10-18y)** |  |
| --- | --- | --- | --- | --- | --- | --- |
|  | % | OR (95%CI) | % | OR (95%CI) | % | OR (95%CI) |
| Overall* |  | N/A | 30·9 |  | 28·9 |  |
| European region |  |  |  |  |  |  |
| North (ref) | 31·9 | 1·00 | 35·3 | 1·00 | 29·9 | 1·00 |
| Central | 28·7 | 0·85 (0·59, 1·21) | 32·4 | 0·85 (0·61, 1·18) | 28·5 | 0·93 (0·56, 1·54) |
| South | 24·3 | **0·66 (0·51, 0·84)** | 26·1 | **0·62 (0·49, 0·77)** | 23·4 | **0·69 (0·51, 0·93)** |

All estimates are adjusted for sex, age, wear time, country, season, study year and ActiGraph models. Study used as cluster variable. *In overall estimates each country weighted by the square root of participants within each country.
